# Metastatic choriocarcinoma in a young woman presenting as thyroid storm: a case report

**DOI:** 10.1186/s13256-021-03123-7

**Published:** 2021-10-23

**Authors:** Muhammad Saleem, Sher M. Sethi, Abrar Ali, Zareen Kiran

**Affiliations:** The Aga Khan University Hospital, Karachi, Pakistan

**Keywords:** Thyroid crises, Thyrotoxicosis, Choriocarcinoma

## Abstract

**Background:**

Thyroid storm is an endocrine emergency and life-threatening condition discovered in 1926. There is no specific laboratory parameter that can differentiate or distinguish between thyroid storm and primary hyperthyroidism. Diagnosis is made on a clinical scoring system, including the Burch–Wartofsky point scale and Japanese Thyroid Association scoring system. The management is early diagnosis, immediate initiation of anti-thyroid medications, intensive care monitoring, and prevention of multiorgan failure.

**Case presentation:**

A 30-year-old Pakistani female presented with complaint of headache, vomiting, and generalized weakness for 3 weeks. She had an episode of seizure-like activity at home, and so was rushed to the emergency department. A detailed thyroid examination revealed a soft, nontender gland with no enlargement or bruit and no exophthalmos. Her thyroid-stimulating hormone was extremely low, with high free triiodothyronine and thyroxine. Thyroglobulin was 425 ng/ml (normal reference range ≤ 55 ng/ml), and thyroid-stimulating hormone receptor antibody was 0.87 IU/L (normal reference range 0–1.75 IU/L). She had high levels of beta-human chorionic gonadotropin hormone on initial presentation. Transvaginal ultrasound showed no intrauterine pregnancy, but an echogenic focus was found adherent to the right ovary with no vascularity. With the chief complaint of headache, she underwent magnetic resonance imaging of the brain that showed multiple scattered hemorrhagic lesions in supratentorial and infratentorial brain parenchyma that were highly suspicious for metastases. Computed tomography scan of the chest, abdomen, and pelvis revealed multiple neoplastic lesions in the lung, liver, spleen, and kidneys. A Tru-Cut liver biopsy showed linear cores of liver tissue with metastatic carcinoma with morphological features consistent with choriocarcinoma. Our patient scored 65 on the Burch–Wartofsky point scale. As per the Japanese Thyroid Association scoring system, our patient met the criteria for a “definite thyroid storm.” She had initiated propranolol to achieve adequate control of her heart rate and dexamethasone. Carbimazole was started to control her thyroid function. Her thyroid function after 2 weeks of treatment showed significant improvement. Methotrexate and etoposide were given for choriocarcinoma. She made a good recovery and was discharged home. She will undergo rehabilitation along with ongoing chemotherapy (methotrexate and etoposide weekly till beta-human chorionic gonadotropin levels normalize). Unless her source of beta-human chorionic gonadotropin is carefully under control, she will continue to take anti-thyroid medications.

**Conclusion:**

Choriocarcinoma is not only associated with hyperthyroidism but can induce thyroid storm. Beta-human chorionic gonadotropin is directly associated with promoting thyroid dysfunction. Patients with gestational trophoblastic disease should be under close surveillance to prevent thyroid storms.

## Background

Thyroid storm is an endocrine emergency and life-threatening condition discovered in 1926 [1]. There is no specific laboratory parameter that can differentiate or distinguish between thyroid storm and primary hyperthyroidism [2]. Diagnosis is made on a clinical scoring system, including the Burch–Wartofsky point scale and Japanese Thyroid Association scoring system [3]. The management is early diagnosis, immediate initiation of anti-thyroid medications, intensive care monitoring, and prevention of multiorgan failure [4]. Here we report a case of thyroid storm in a patient with metastatic choriocarcinoma.

## Case presentation

A 30-year-old Pakistani female, married, mother of two children, presented to the emergency department in May 2021 with complaint of headache, vomiting, and generalized weakness for 3 weeks. She had an episode of seizure-like activity at home, and so was rushed to the emergency department. Headache is defined as generalized pain mainly over the vertex that has gradual onset and is dull in nature, nonradiating, intermittent in duration with days going through worst pain followed by days without pain, moderate in severity ranging from 7 to 8 on a pain scale, and associated with nausea, vomiting, and generalized weakness. It was only temporarily relieved with pain medications. There was no stress precipitant in her life to induce a headache. The single episode of seizure-like activity included up-rolling of eyes and drooling of saliva, with no generalized recognizable altered limb movements and no urinary or fecal incontinence. It was followed by a few minutes of unconsciousness. After gaining consciousness, she exhibited confusion and altered mental status. She also had an undocumented fever, without chills and rigors, which was relieved with antipyretic. The patient denied any neck stiffness, irrelevant behavior and mood changes, vision alteration, excess lacrimation, and photo- or phonosensitivity. In addition to this, she lost her appetite and felt like she was losing weight, although her bowel and urine habits were usual. The rest of the systemic review was unremarkable. Her past medical history was significant for two cesarean sections, with the last-born child in January 2021. She had no menstruation after the delivery. Occasionally, she took paracetamol (acetaminophen) for pain relief and fever. She negated smoking or any other addictions. There was no history of allergies to particles, food, or any medications.

On physical examination, she was agitated and acutely anxious but still awake, alert, and oriented to time, place, and person. Her Glasgow Coma Scale score was 15 over 15. Initial blood pressure was 138/72 mmHg, heart rate was 105 beats per minute, respiratory rate was 22 breaths per minute, and temperature was 38.0 °C. Jugular venous pressure was not distended, and there was no appreciable peripheral edema and lymph node enlargement. Cardiac examination showed tachycardia with a regular pulse, and there were no added sounds. Respiratory examination revealed distant air entry in bilateral lung fields. No crepitation or wheezes were noted. Abdominal examination was insignificant with no organomegaly. Neurological examination depicted confusion with normal speech, delay to response, equally reactive pupils, intact cranial nerves, no motor or sensory deficit, normal reflexes, and down-going plantar response. A detailed thyroid examination revealed a soft, nontender gland with no enlargement or bruit and no exophthalmos. Gynecological examination revealed a horizontal scar in the pelvis of the previous C-section, with normal external genitalia and no vaginal mass, discharge, or atrophy on speculum examination. The uterus was anteverted and had multiple blood clots in the cervical canal.

Based on history and examination, she underwent an extensive investigation to identify the cause of her illness. Due to the ongoing pandemic, severe acute respiratory syndrome coronavirus 2 (SARS-CoV-2) antigen test and polymerase chain reaction were done, which came back negative. Initial complete blood count and metabolic panel were insignificant (Table 1).

**Table 1:** Initial laboratory parameters of the patient

	Value	Reference range
Hemoglobin	10.1 g/dl	11–14.5
White cell count	8.2 × 10^9^/L	4.6–10.8 × 10^9^
Platelets	223 × 10^9^/L	154–433 × 10^9^
Creatinine	0.7 mg/dl	0.6–1.1
Sodium	132 mmol/L	136–145
Potassium	3.9 mmol/L	3.5–5.1
Bicarbonate	22.7 mmol/L	20–31
Calcium	8.6 mg/dl	8.6–10.2
Magnesium	1.8 mg/dl	1.6–2.6
SGPT/ALT	67 IU/L	< 35
SGOT/AST	114 IU/L	< 31
International normalized ratio	1.0	0.9–1.2
C-reactive protein	32.39 mg/L	0–14
Procalcitonin	0.219 ng/ml	> 2
TSH	< 0.010 uIU/ml	0.4–4.2
Free T4	4.74 ng/dl	0.89–1.76
Free T3	17.90 pg/ml	2.1–4.4
Beta-hCG	3,058,000 IU/ml	Nonpregnant: < 10
Troponin I (initial)	1.485 ng/ml	> 0.04
Troponin I (after 6 hours)	0.856 ng/ml	–

SGPT/ALT: Serum glutamic pyruvic transaminase/Alanine aminotransferase; SGOT/AST: Serum glutamic oxaloacetic transaminase/Aspartate aminotransferase; TSH: thyroid stimulating hormone; Beta-hCG: Beta-human chorionic gonadotropin

**Fig. 1:**
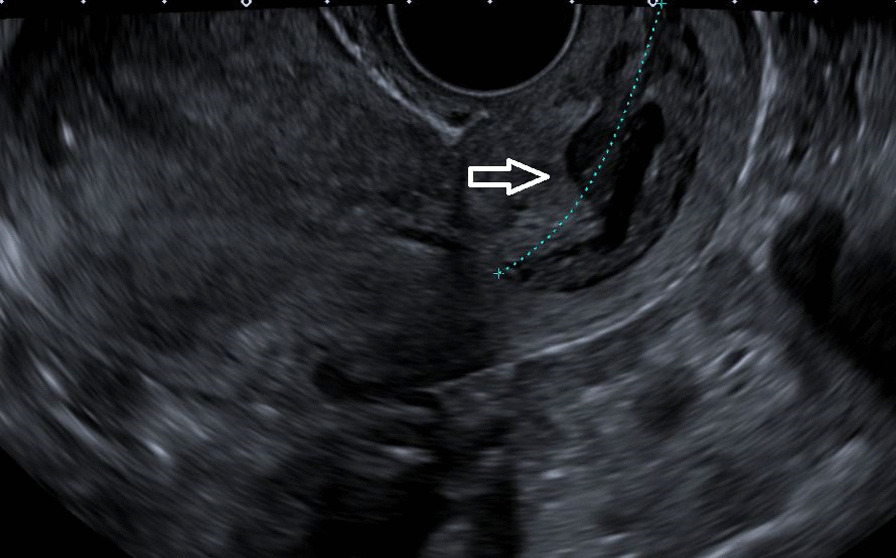
Transvaginal ultrasound showing an echogenic focus (shown by white arrow) adherent to right ovary with no vascularity

**Fig. 2:**
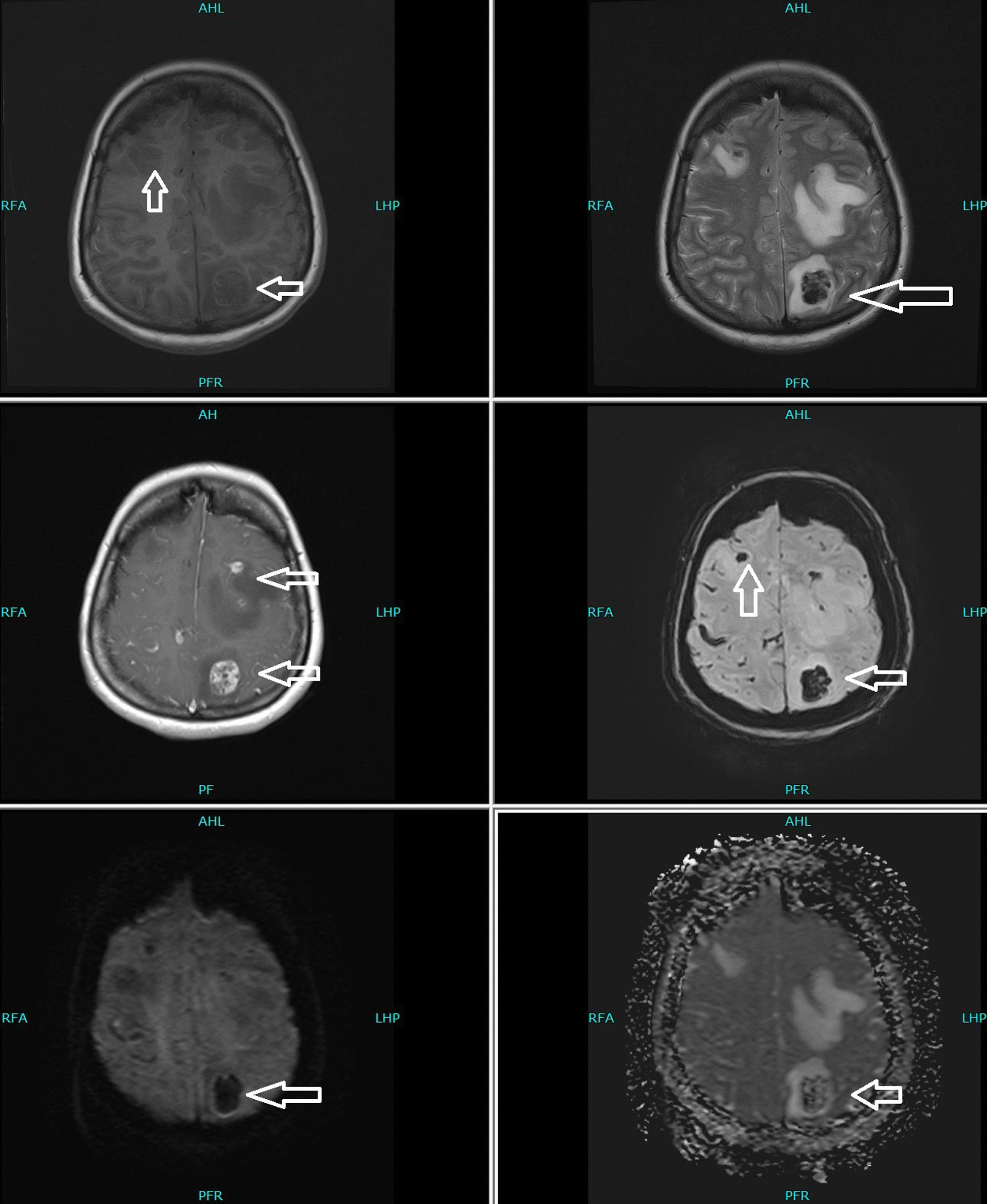
Magnetic resonance imaging of the brain showing scattered hemorrhagic lesions in supratentorial and infratentorial brain parenchyma as shown with white arrows

**Fig. 3:**
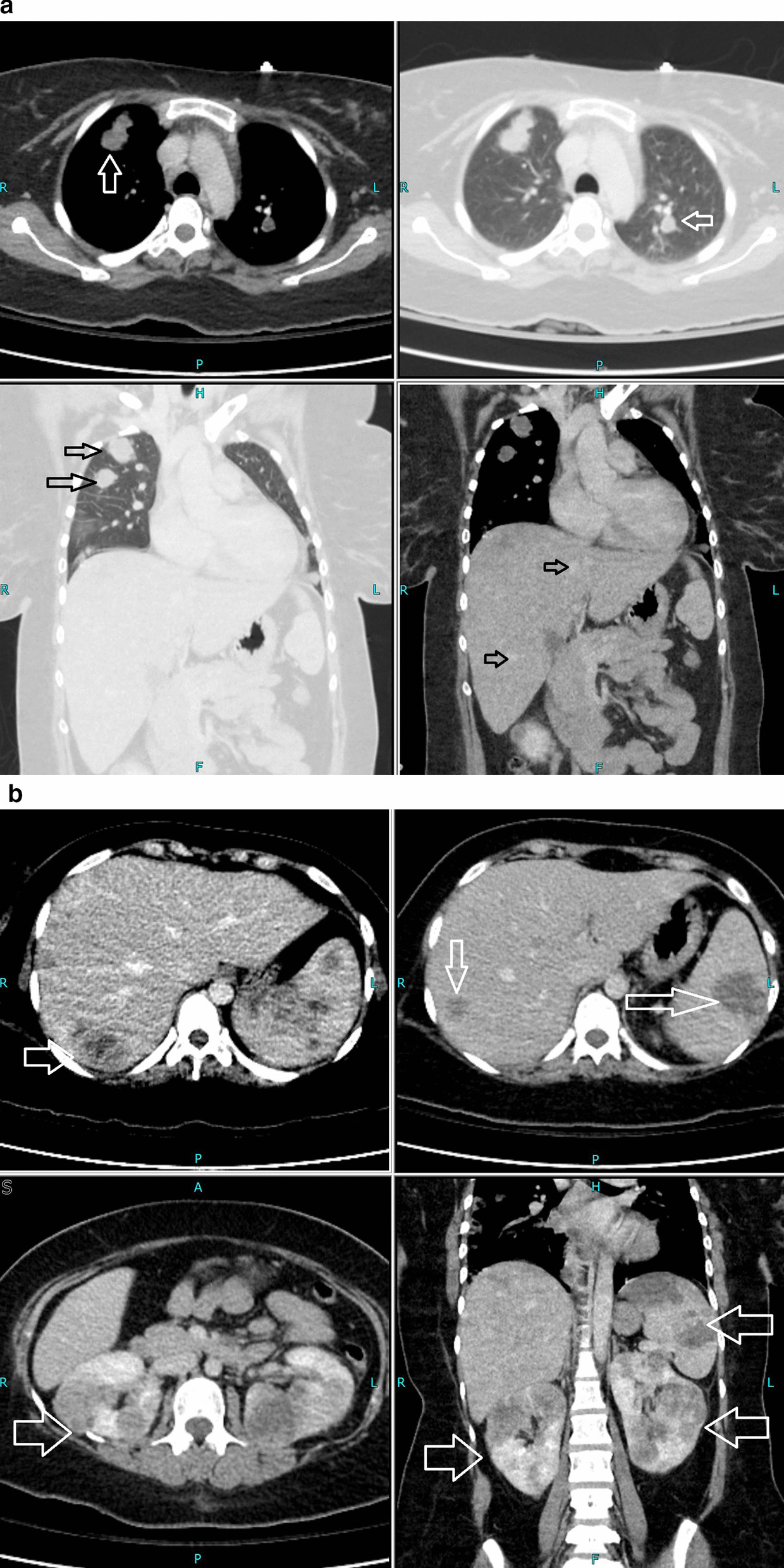
**a** Computed tomography of the chest showing neoplastic lesions in the lung (white and black arrows). **b** Computed tomography of the abdomen and pelvis (white and black arrows) showing multiple neoplastic lesions in liver, spleen, and kidneys

Her thyroid-stimulating hormone (TSH) was extremely low, with high free triiodothyronine (T3) and thyroxine (T4), warranting us to perform more investigations to identify the cause. Thyroglobulin was 425 ng/ml (normal reference range ≤ 55 ng/ml), and TSH receptor antibody (TRAb) was 0.87 IU/L (normal reference range 0–1.75 IU/L). Due to high levels of beta-hCG hormone on initial presentation, she underwent a transvaginal ultrasound that showed no intrauterine pregnancy but an echogenic focus adherent to the right ovary with no vascularity. The finding could be secondary to ectopic pregnancy versus neoplastic lesion (Fig. 1). With the chief complaint of headache, she underwent magnetic resonance imaging (MRI) of the brain that showed multiple scattered hemorrhagic lesions in supratentorial and infratentorial brain parenchyma that were highly suspicious for metastases with an unknown primary (Fig. 2). Computed tomography (CT) scans of the chest, abdomen, and pelvis were done to identify the primary tumor and for the staging of the disease. The scan revealed multiple neoplastic lesions in the lung, liver, spleen, and kidneys (Fig. 3a, b). She then underwent a Tru-Cut liver biopsy that showed linear cores of liver tissue with metastatic carcinoma with morphological features consistent with choriocarcinoma.

Thyroid storm was diagnosed based on the clinical scoring system proposed by Burch and Wartofsky, which is the most widely used [5]. Our patient scored 65 on the Burch–Wartofsky point scale (Table 2). A score of more than 45 is highly suggestive of thyroid storm. As per the Japanese Thyroid Association scoring system [6], our patient met the criteria for “definite thyroid storm” with thyrotoxicosis and one feature of the central nervous system (seizures) along with fever and gastrointestinal manifestations.

**Table 2:** Burch–Wartofsky point scale (BWPS)

	Patient parameters	Point scores
Temperature (°C)	38 °C	10 points
Central nervous system manifestation	Seizures	30 points
Gastrointestinal manifestation	Nausea/vomiting	10 points
Heart rate	105 beats per minute	5 points
Congestive heart failure	Absent	0 points
Atrial fibrillation	Absent	0 points
Precipitating events	Present	10 points
		Total score = 65 points

**Fig. 4:**
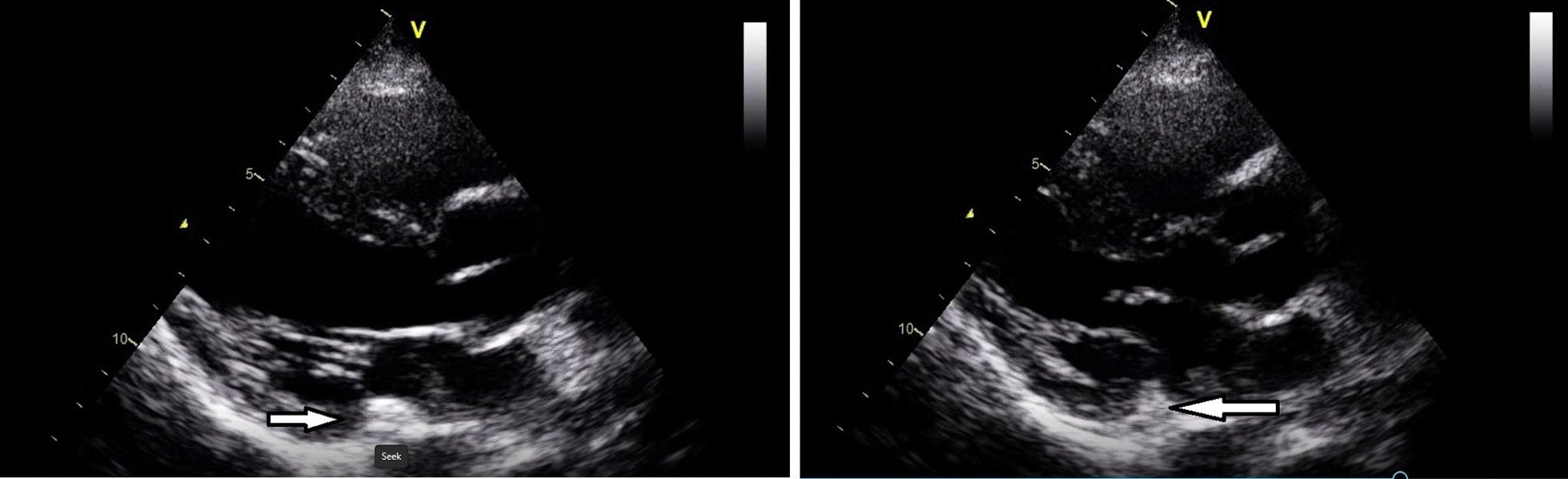
Echocardiography showing mobile, finger-like echogenic density in left atrium shown with white arrow

The patient was initially admitted to a special care unit, but later she developed generalized tonic–clonic seizures that were not aborted with diazepam and antiepileptic medications. The patient went into status epilepticus and so was electively intubated, was sedated with propofol and midazolam, and was shifted to the intensive care unit. The oncology team was called and advised of additional work-up before proceeding towards chemotherapy. Her serum alpha-fetoprotein was 2.0 IU/ml (normal reference range < 6.7 IU/ml), whereas human immunodeficiency virus (HIV) serology and hepatitis B and C came out to be nonreactive. Interestingly, echocardiography was done showing preserved ejection fraction (60%) with no segmental wall motion abnormality. A mobile, finger-like echogenic density was noted in the left atrium, probably attached to the pulmonary vein, which is suggestive of thrombus or mass or vegetation (Fig. 4). Her blood cultures were negative, and there was no history of joint pain and rash, making infective endocarditis less likely. The cardiology team was called, and they considered it as a part of ongoing systemic disease. Her creatinine clearance was 73 ml/minute (normal reference range 65–123 ml/minute). For choriocarcinoma, she was started on multiple chemotherapy regimen of methotrexate and etoposide.

Endocrine team was consulted during the intensive care unit (ICU) stay. She had already been initiated on propranolol 20 mg every 8 hours to achieve adequate control of her heart rate and dexamethasone 10 mg every 8 hours by the ICU team. She was also started on carbimazole 15 mg every 12 hours. She was then started on cholestyramine 4 g every 6 hours to help bind and excrete excess thyroid hormone and Lugol’s iodine solution (ten drops every 8 hours). The results of her thyroid function test repeated after 2 weeks of treatment are presented in Table 3.

**Table 3:** Thyroid profile of the patient

	Initial	After treatment (2 weeks)	Reference range
TSH	< 0.010 uIU/ml	< 0.010 uIU/ml	0.4–4.2
Free T4	4.74 ng/dl	1.61 ng/dl	0.89–1.76
Free T3	17.90 pg/ml	3.40 pg/ml	2.1–4.4

TSH: thyroid stimulating hormone

During ICU stay, her Glasgow Coma Scale (GCS) score dropped from 15/15 to 5/15, so urgent CT of the head was done, which showed diffuse cerebral edema with midline shift. Neurosurgery team was called, and she was started on hypertonic saline. Later, she underwent decompressive craniotomy for her brain lesion to reduce mass effect and tracheostomy for prolonged respiratory support. She was then transitioned to a portable ventilator, which she tolerated well and so was shifted out of the intensive care unit.

She made a good recovery and was gradually tapered off the sedation. Antiepileptic medications were further optimized by the neurology team, and she was gradually weaned off the ventilator. She was successfully discharged home and planned to undergo rehabilitation along with ongoing chemotherapy (methotrexate and etoposide weekly till beta-hCG level normalize). Unless her source of beta-HCG is carefully under control, she will continue to take anti-thyroid medications.

## Discussion

We report here an interesting case of a middle-aged female with metastatic choriocarcinoma presenting with thyroid storm.

Thyroid storm is a rare and life-threatening endocrine emergency. Mortality with thyroid storm is around 30%. It is considered to be precipitated by physiological stress caused by surgery, trauma, infections, metabolic acidosis, and uncontrolled hyperthyroidism [7]. Pregnancy and childbirth are also considered triggering factors for thyroid storms [8]. Our patient also had a recent cesarean section, making her prone to having thyroid storm.

The clinical features used for diagnosing thyroid storm include fever, tachycardia, central nervous system (CNS) manifestation, gastrointestinal (GI) manifestations, cardiac manifestations, and precipitating events [5, 6]. Fever and tachycardia were present in our patient. CNS manifestations include altered mental status, agitation, psychosis, seizures, and coma. GI manifestations include jaundice, nausea, vomiting, and abdominal pain [9]. Our patient had a seizure that was not aborted by antiepileptics. This could be an overlapping symptom between thyroid storm or brain metastasis. She had vomiting with mild hepatic dysfunction.

Pituitary tumors secreting thyroid-stimulating hormone are also unusual. They usually present with hyperthyroidism. Fujio *et al.* reported a case from Japan of thyroid storm with TSH-secreting pituitary malignancy [10]. Various case reports show an association between gestational trophoblastic disease and thyroid storm. Females of childbearing age are at high risk and should be evaluated to prevent fatal outcomes [11, 12]. Here, we also report a case of disseminated gestational trophoblastic disease presenting with thyroid crises.

Gestational trophoblastic disease (GTD) is a group of disorders that includes benign conditions (such as hydatidiform mole) and malignant conditions (such as invasive mole, choriocarcinoma, placental site, and epithelioid trophoblastic tumor) [13]. The pathophysiology of thyroid disease with gestational trophoblastic disease is secretion of beta-human chorionic gonadotropin (hCG) hormone by trophoblastic tissue. The two known main mechanisms of thyroid disorder with increased beta-hCG are enhanced thyrotropic activity by beta-hCG and structural resemblance with TSH causing a release of thyroxine from the thyroid gland [14]. Kofinas *et al.* reported a beta-hCG level in her patient with a thyroid storm of around 1,488,021 IU/ml [11]. Our patient had a very high level of beta-hCG (3,058,000 IU/ml), and the above-mentioned mechanism explains the cause of thyroid storm in her.

A choriocarcinoma is a malignant form of GTD. Kato *et al.* reported an incidence of hyperthyroidism with choriocarcinoma of approximately 57%, while in normal pregnancy it is 18%. This was associated with high levels of beta-hCG as compared with normal pregnancy [15]. A recent case report had been published of a patient with testicular choriocarcinoma presenting with thyroid storm [16]. Morley *et al.* reported three cases of metastatic choriocarcinoma with thyrotoxicosis. They also postulated that higher levels of beta-hCG are associated with an increased risk of thyroid storm [17]. Sotello *et al.* reported metastatic choriocarcinoma in men with thyrotoxicosis [18]. Similarly, our patient was also diagnosed with metastatic choriocarcinoma along with thyroid storm. Interestingly, testicular choriocarcinoma was more associated with thyroid crises. In our case, the patient was a woman of childbearing age with a right ovary lesion and metastasis to the brain, lungs, liver, and kidneys.

Future observational studies are required for identifying the incidence and prevalence of thyroid storm with GTD. Though hyperthyroidism is common with GTD, study of its outcomes in these individuals has been limited. We encourage endocrinologists and gynecologists to collaborate and formulate a mechanism for early identification, proper management, and assessment of outcomes of thyroid storm presenting in patients with gestational trophoblastic disease.

## Conclusion

Choriocarcinoma is not only associated with hyperthyroidism but can induce thyroid storm. Beta-hCG is directly associated with promoting thyroid dysfunction. Patients with gestational trophoblastic disease should be monitored closely to prevent thyroid storms.

## Data Availability

Can be reviewed on editor’s request.
